# Intended Ranges and Correlations between Percentages of Variables Like Oleic Acid, Eicosapentaenoic Acid, and Arachidonic Acid

**DOI:** 10.3390/foods10051012

**Published:** 2021-05-06

**Authors:** Arne Torbjørn Høstmark

**Affiliations:** Faculty of Medicine, Institute of Health and Society, University of Oslo, P.O. Box 1130 Blindern, 0318 Oslo, Norway; a.t.hostmark@medisin.uio.no; Tel.: +47-22844629; Fax: +47-22850590

**Keywords:** oleic acid, arachidonic acid, eicosapentaenoic acid, random numbers, human sera, chicken muscle

## Abstract

In chicken muscle, we previously showed that ranges of oleic acid (OA), arachidonic acid (AA), and eicosapentaenoic acid (EPA) might explain why %OA was inversely related to %AA, and that %EPA correlated positively with %AA. We here try to clarify further how ranges of the fatty acids could make strong associations between their relative amounts, utilizing published data from chicken muscle and human sera. We generated random number variables (OA*’*, AA*’*, EPA*’*) in lieu of the true variables, and we studied effects of altering their ranges upon scatterplots of %OA*’* vs. %AA*’* (%EPA*’*), and %AA*’* vs. %EPA*’*. To explain the results, we first applied the equation OA*’* + AA*’* + EPA*’* = S, i.e., %OA*’* + %AA*’* + %EPA*’* = 100. Next, we considered how the OA*’* (AA*’*, EPA*’*) fractions of S related to S. Increasing the OA*’* range towards higher values improved the positive association between %AA*’* and %EPA*’*. Thus, increased intake of OA could improve the positive correlations between percentages of eicosanoid precursors, raising the question of whether *“intended ranges”* of some fatty acids represent a case of evolutionary selection to, e.g., achieve balance between eicosanoids.

## 1. Introduction

Oleic acid (OA, 18:1 c9) and foods rich in OA, e.g., olive oil, may positively influence insulin sensitivity, endothelium-dependent flow-mediated vasodilatation [1], serum LDL and HDL cholesterol [2,3,4], blood pressure [5], and may have anti-carcinogenic and anti-inflammatory effects [6,7,8]. Additionally, oleic acid may cause LDL to be less prone to be oxidized [9,10]. 

Furthermore, olive oil might decrease the concentration of arachidonic acid (AA, 20:4 n6), which is synthesized from linoleic acid (18:2 n6), and metabolized further into various eicosanoids, i.e., prostacyclin, thromboxane, leukotrienes, and lipoxins [11]. Thromboxane A_2_ (TXA_2_) and leukotriene B_4_ (LTB_4_) stimulate inflammatory and thrombotic reactions [12,13]. In addition, EPA and DHA (docosahexaenoic acid) are precursors of resolvins and protectins, which are components appearing during the initiation and resolution phases of inflammatory reactions. Docosanoids such as protectins, resolvins, and maresins originate from C22 fatty acids, i.e., docosapentaenoic acid (DPA) and DHA. Some docosanoids may strongly counteract immune and inflammatory reactions [11]. Furthermore, endocannabinoids, derived from AA, may have a role in adiposity and inflammation [14].

We previously observed that %OA related inversely to %AA in chicken muscle [15], and in sera of humans [16], and rats [17] Possibly, feedback regulation between the formation of OA and AA might be one explanation of the inverse relationship [15,16,17]. 

EPA and AA are metabolic antagonists [11,13,18]. Eicosanoids derived from EPA may beneficially influence inflammatory diseases [19,20], improve coronary heart diseases [21,22], and cancer [23]. Accordingly, we should expect a balanced metabolic regulation of EPA and AA percentages t, i.e. %EPA and %AA should increase (decrease) simultaneously. However, some alleged positive health effects of long-chain n3 fatty acids have been questioned [24].

In line with the reasoning above, we recently found a positive correlation between %AA and %EPA in breast muscle lipids of chickens [25,26]. Furthermore, the association seemed to depend on the particular range (g/kg wet weight) of EPA and AA. Additionally, percentages of other precursors of eicosanoids (docosanoids) were positively associated as well, as observed in breast muscle lipids of chickens [25,26,27,28,29]. Moreover, we were able to reproduce the positive correlations with random numbers, if the numbers had the true ranges of the fatty acids. This finding raises the question of whether evolution might have selected intended ranges for some fatty acids. The aim of the present work was to clarify how intended ranges of OA, AA, and EPA could make strong associations between their relative amounts, with particular reference to the OA range.

## 2. Materials and Methods

To clarify further how correlations between relative amounts of OA, AA, and EPA might arise, in this study we used random numbers only and marked the substitute, random number variables OA’, AA’, and EPA’. Our previous studies [25,26,27,28] showed similar correlations between relative amounts of OA, AA, and EPA, and between OA’, AA’ and EPA’, on the condition that ranges of the random number variables were like the corresponding true ranges. To clarify that we used random numbers, in the figure texts we write RANDOM in uppercase letters.

In the computer experiments, we applied previously reported values of fatty acid concentrations, as found in chicken breast muscle [28], and in human sera [30]. To generate the random numbers, we utilized the reported mean ± SD values, and/or ranges of OA, EPA, and AA. Next, we computed S = OA’ + EPA’ + AA’. Percentages of the variables were computed as %AA’ = (AA’/S) × 100; %EPA’ = (EPA’/S) × 100; %OA’ = (OA’/S) × 100. Two approaches were used to study how the percentages might correlate: (1) utilizing the equation of a straight line (y = ax + b), and (2) studying the association between S and OA’ (AA’, EPA’) fractions (percentages) of S. The equation %OA’ + %AA’+ %EPA’ = 100 may be rewritten to (1) %AA’ = − %EPA’ + (100 − %OA’), (2) %OA’ = − %AA’ + (100 − %EPA’), and (3) %OA’ = − %EPA’ + (100 − %AA’). In the computer experiments, we studied the effect of altering the ranges of OA’, EPA’, and AA’ upon associations between their relative amounts. The random numbers had either uniform distribution (sampled from ranges), or normal distribution (generated based on the mean (± SD) values). The order of magnitude of the current fatty acids was similar in chicken muscle [28] and in human sera [30], i.e., being OA > AA > EPA, and OA >> (AA + EPA). For simplicity, in all of the computer experiments below, we arbitrarily generated 200 random number “cases”. Thus, in the analyses we had 200 values of S, and of S-fractions (percentages). To illustrate distributions of the percentages, we made histograms. Thus, we generated random numbers for OA, AA, and EPA with (1) their physiological ranges, and (2) with hypothetical ranges. We made scatterplots of %AA’ vs. %EPA’, and of %OA’ vs. %AA’ (%EPA’). Spearman’s rho was used to study correlations. The two approaches used to explain the results are described in more detail under Results and Discussions, where we first present some theoretical considerations, and then show results of computer experiments to test suggestions made from the theory.

We conducted many repeats of the analyses, each with a new set (*n* = 200) of random numbers. The corresponding outcomes were always very similar; scatterplots appeared unchanged, but corresponding correlation coefficients varied slightly. We used SPSS 27.0 for the analyses and for making figures. The significance level was set at *p* < 0.05.

## 3. Results and Discussions

### 3.1. General Considerations

We previously [15] tried to explain associations between %OA, %AA, and %EPA, utilizing the equation of a straight line. The present work is an extension, where we additionally used the *“*relation-to-sum*”* approach (see below, and Methods). It seems pertinent to present briefly some of the previous theoretical consideration, however using new sets of random numbers in the computer tests.

### 3.2. Applying the Equation of a Straight Line (y = ax + b)

If S is the sum of many positive scale variables, S = A + B + C + …, we may simplify to S = A + B + R, i.e., %A + %B + %R = 100, or %B = −%A + (100 − %R), where R is the sum of all variables, except A and B. This equation seems to resemble the equation of a straight line, however involving percentage amounts of three unknown variables (A, B, R), each of which with a defined range. We consider two hypothetical extreme conditions: (1) the expression (100 − %R) approaches zero, and (2) %R approaches zero.

### 3.3. %R Close to 100

#### 3.3.1. The %A vs. %B Association

If %R consists of very high values and (100 − %R) > %A, the equation appears to approach %B = %A, apparently showing a linear, positive association between %A and %B. The requirement (100 − %R) > %A is satisfied, since the remaining value when calculating (100 − %R) would have to be divided between %A and %B. Hence, the slope of the %A vs. %B regression line should be positive. We may estimate the slope by utilizing maximum and minimum values of %B and %A, i.e., by the ratio

(%B_max_ − %B_min_)/(%A_max_ − %A_min_). A more general equation would therefore be

%B_(p–q)_ = [(%B_max_ − B_min_)/(%A_max_ − %A_min_)]  · %A _(r–s)_ + z (1)

Ranges of %A and %B are shown in subscript parentheses, and z = 100 − %R. 

#### 3.3.2. The Correlation between %R and %A (%B)

Since %R has very high values, %A and %B should be small. We rewrite the equation %A + %B + %R = 100, to be %B = −%R + (100 − %A). With very small %A-values, the equation would approach %B = −%R + 100, suggesting that %R and %B are inversely related. Similarly, the approximation %A = −%R + 100 suggests an inverse %R vs. %A association, and t a negative correlation between %R and %A (%B).

#### 3.3.3. Computer Test

We arbitrarily chose A 1.0–1.3, B 2.0–2.2, R 30–200, to make high values of %R. There was a strong positive association between %A and %B, and a strong negative relationship between %R and %A (%B). Spearman*’*s rho = 0.983 for %A vs. %B; rho = −0.992 (−0.998) for %R vs. %A (%B), *p* < 0.001 for all, *n* = 200. Quartiles of %A, %B and %R were 0.8, 1.1, 1.6; 1.4, 1.9, 2.9; and 95.5, 97.1, 97.9, respectively. Thus, %R had high values relative to %A and %B. Skewness of %A, %B, and %R was 1.26, 1.24, and −1.24, respectively (SD 0.17 for all). We previously explained this skewness outcome.

### 3.4. %R Close to Zero

With very low values of %R in the equation %B = −%A + (100 − %R), we would expect a negative %A vs. %B association, since the equation in this case would approach %B = −%A + 100. However, in this case we should probably not expect that a decrease in %R would suffice to compensate a major increase in %A or %B. Hence, we should probably expect a poor correlation between %R and %A (%B).

Computer test: To obtain very low values of %R relative to %A and %B, we arbitrarily chose A 10–50, B 20–67, R 0.10–0.13. Spearman*’*s rho = −1.000 for %A vs. %B, *p* < 0.001, *n* =200; rho = 0.044 (−0.048), *p* = 0.532 (0.502) for %R vs. %A (%B). Quartiles of %A, %B and %R were 33.2, 40.8, 50.0; 49.9, 59.1, 66.6; 0.12, 0.15, and 0.18, respectively. Thus, values of %R were small relative to those of %A and %B.

### 3.5. Considering how Sum (S) of the Variables Relates to Their Fractions of S

We limit our reasoning to positive scale variables. With a combination of two low-number variables (A, B) having narrow ranges relative to a third one (R), we might expect a positive association between %A and %B, and a negative relationship between %R and %A (%B).

### 3.6. Two Positive Scale Variables (A and B) with Narrow Ranges Relative to a Third One (R) with High Variability

To explain the correlation outcome, we omit ranges of the variables and write A + B + R = S. The A, B, and R fractions of S are Af = A/S, Bf = B/S, and Rf = R/S, respectively. Thus, Af = A/(A + B + R) = 1/(1 + B/A + R/A). However, since we—in the current context—define ranges of A and B to be very narrow, the B/A ratio is close to be a fixed number. Therefore, Af would approach Af = 1/(t +R/A) where t approaches a constant, i.e., t = 1 + B/A. Similarly, the B-fraction of S, Bf = B/(A + B + R) = 1/(1 + A/B + R/B), i.e., Bf = 1/(k + R/B), where k is close to be a constant: k = (1 + A/B). This means that R will largely govern the A (B) fractions (percentages) of S. Thus, when R and S (being mainly composed of R) go from lowest to highest value, then Af = 1/(t + R/A), and also Bf = 1/(k + R/B) will go from the highest to the lowest value. Hence, S should relate inversely to the A and B fractions (percentages). Accordingly, we should expect percent A to be positively associated with %B. However, increasing the A-and/or B-ranges (variabilities), and/or decreasing the R-range, would cause deviations from the above restrictions, and accordingly change the %A vs. %B association, to be reflected in altered scatterplots and correlation coefficients.

The R-fraction of S is Rf = R/S = R/(A + B + R), i.e., Rf = 1/(1 + z/R), where z is close to a constant, z = A + B. Therefore, the R fraction (and percentage) of S should increase with increasing R (from lowest to highest value), and accordingly also with increasing S, because R is the main contributor to S. Thus, S should be positively associated with %R. It follows that %R should be negatively associated with %A and %B.

Computer test: To achieve A and B with narrow ranges relative to R, we arbitrarily chose A 3.00–3.13, B 7.00–7.16, R 6–47, and generated 200 uniformly distributed random numbers with these ranges. As expected, S correlated negatively with %A (%B), i.e., rho = −0.999 (−1.000), and positively with %R (rho = 1.000), *p* < 0.001 for all. Accordingly, %A correlated positively with %B (rho = 0.999), and %R was negatively associated with %A (%B), rho = −0.999 (−1.000).

### 3.7. Two Positive Scale Variables (A and B) with Broad Ranges (High Variability) Relative to a Third One (R) with Low Numbers and Very Low Variability

When approaching a condition with two variables only, their relative amounts should relate negatively. The A-fraction of S, Af = A/(A + B + R) = 1/[1 + (B +R)/A], should increase as B goes from highest to lowest value, and/or A goes from lowest to highest value. Similarly, the B-fraction of S, Bf = B/(A + B + R) = 1/[1 + (A + R)/B], should increase as A runs from highest to lowest value, and/or B runs from lowest to highest value. Since Bf decreases as Af increases, we should expect a negative association between %A and %B, in the current case.

Computer test: To obtain broad ranges of A and B, and low numbers and narrow range of R, we arbitrarily chose: A 3–43; B 7–89; R 0.10–0.12, emphasizing that we chose these values just to illustrate a mathematical point, without any relationship to biology. In line with the above reasoning, %A was negatively associated with %B, rho = −1.000, *p* < 0.001, *n* = 200. Correlations between %R and %A (%B) were poor: rho = 0.399 (−0.402), *p* < 0.001, *n* = 200.

#### Slope of the %A vs. %B Regression Line

The equation %B = −%A + (100 − %R) seems to resemble the equation of a straight line. The slope (ΔY/ΔX) of the regression line for the %A vs. %B association may be roughly estimated using maximum and minimum values of the A and B percentages, i.e., ΔY/ΔX = (%B_max_ − %B_min_)/(%A_max_ − %A_min_). With ranges added, the equation would be

%B_(p–q)_ = [(%B_max_ − %B_min_)/(%A_max_ − %A_min_)] * %A _(r–s)_ + z (2)

Subscript parentheses indicate ranges of %A and %B, and z = 100 − %R. Thus:

ΔY/ΔX = (100·B_max/_S_min_ − 100·B_min/_S_max_)/(100·A_max/_S_min_ − 100·A_min/_S_max_) (3)

Since we define ranges of A and B to be very narrow, we may do the following approximations:

A_max_ = A_min_ = **A**, and B_max_ = B_min_ = **B**, giving ΔY/ΔX = (**B·**S_max_ − **B·**S_min_)/(**A·**S_max_ − **A·**S_min_) = **B/A** (4)

Accordingly, the **B/A** ratio may estimate the slope, if the ranges of **A** and **B** are very narrow relative to that of R.

### 3.8. Oleic Acid Range and Correlations between Relative Amounts of OA, AA, and EPA

With reference to the presented general considerations, below we reason further about how %OA, %AA, and %EPA should relate, and how alterations in ranges might influence the associations.

### 3.9. Considering Reported Data from Chickens

In chicken breast muscle [28], ranges of AA and EPA were narrow, and with low numbers, i.e., 0.2–0.3 g/kg for AA, and 0.1–0.2 g/kg for EPA, as compared with the broad OA-range (1–9 g/kg), i.e., we have two variables with narrow ranges (EPA and AA) relative to that of OA.

Applying Approach 1: If S = OA + AA + EPA, we may write %OA + %AA + %EPA =100, or %AA= −%EPA + (100 − %OA). Since ranges of EPA and AA are narrow relative to that of OA, we should expect a positive association between %AA and %EPA, as explained above. We rewrite the equation to %OA= −%EPA + (100 − %AA), and to %OA= −%AA + (100 − %EPA). Because %EPA and %AA levels are small relative to those of %OA, the equations would approach %OA= −%EPA + 100, and %OA= −%AA + 100, suggesting a negative relationship between %OA and %AA (%EPA).

Applying Approach 2: Since AA and EPA have narrow ranges relative to OA, we would expect that S varies inversely with %AA (%EPA), and positively with %OA. Hence, %A and %B should correlate positively, and %OA should relate negatively to %AA (%EPA).

**Figure 1:**
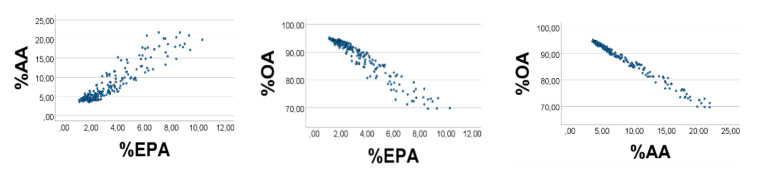
Associations between relative amounts of EPA*’*, AA*’*, and OA*’*, in a computer experiment with RANDOM numbers, see text. S = OA*’* + EPA*’* + AA*’*. RANDOM numbers (*n* = 200) were used in lieu of true values; however, ranges were like those found in chicken muscle, i.e., 0.1–0.2 for EPA*’*, 0.3–0.4 for AA*’*, and 1–9 for OA*’*. %EPA*’* vs. %AA*’*: rho =0.890; %OA*’* vs. %EPA*’* (%AA*’*): rho = −0.951 (−0.984), *p* < 0.001 for all. Note: In the figure we have omitted the random number mark (*‘*) on the variables.

**Figure 2:**
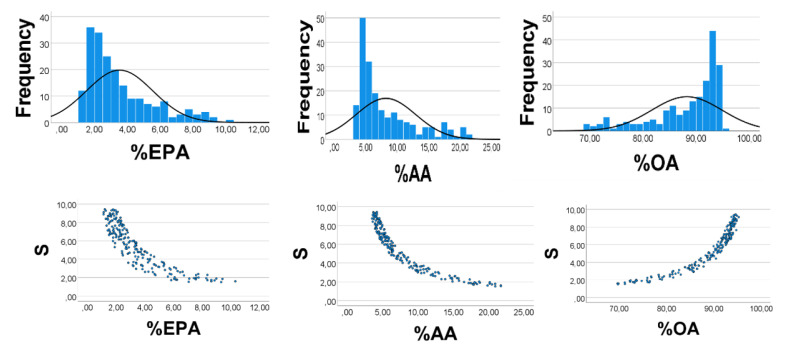
Computer experiment to elucidate associations between relative amounts of OA*’*, EPA*’*, and AA*’*, see text. S = OA*’* + EPA*’* + AA*’*. RANDOM numbers (*n* = 200) were used instead of true values; however, ranges were like those found in chicken muscle, i.e., 0.1–0.2 for EPA*’*, 0.3–0.4 for AA*’*, and 1–9 for OA*’*. Upper panels: Frequency distributions of %EPA*’*, %AA*’*, and %OA*’*. Cutoff values of %EPA*’*, %AA*’*, and %OA*’* quartiles were 2.1, 2.9, 4.4; 3.6, 4.9, 7.4; 88.5, 92.3, 94.4%, respectively. Skewness of the %EPA*’*, %AA*’*, and %OA*’* histograms were 1.42, 1.43, and −1.34, respectively (SE = 0.17 for all). Lower panels: Scatterplot of S vs. % EPA*’* (% AA*’*, %OA*’*). Spearman*’*s rho = −0.915 for S vs. %EPA*’*; −0.956 for S vs. %AA*’*; and 0.967 for S vs. %OA*’*, *p* < 0.001 for all. Note: In the figure we have omitted the random number mark (*‘*) on the variables.

Computer test: We generated uniformly distributed random numbers (*n* = 200) with the reported [28] ranges (g/kg wet weight) of the fatty acids, i.e., OA 1–9, AA 0.3–0.4, EPA 0.1–0.2. As expected, %EPA*’* was positively associated with %AA*’* (Figure 1, left panel), Spearman*’*s rho = 0.890; equation of the regression line was %AA*’* = 2.16 (0.06) %EPA*’* + 0.65 (0.25). Applying the AA*’*/EPA*’* ratio to estimate the slope of the %AA*’* vs. %EPA*’* association, we found the slope to be 0.35/0.15 = 2.3, i.e., not far from the value obtained from the regression line. %OA*’* correlated negatively with %EPA*’* (%AA*’*): rho = −0.951 (−0.984), *p* < 0.001 for all. Skewness of the histograms (Figure 2, upper panels) of %EPA*’*, %AA*’*, and %OA*’* were 1.42, 1.43, and −1.34, respectively (SE = 0.17 for all). Quartiles of the distributions of %EPA*’*, %AA*’*, and %OA*’* were 2.1, 2.9, 4.4; 3.6, 4.9, 7.4; 88.5, 92.3, 94.4, respectively. The low values of %EPA*’* (%AA*’*) as compared with those of %OA*’* might explain the positive %EPA*’* vs. %AA*’* association, and the negative %OA*’* vs. %AA*’* (%EPA*’*) relationship, as explained above.

As shown in Figure 2 (lower panels), S had a curvilinear inverse association with %AA*’* (rho = −0.912) and %EPA*’* (rho = −0.975), but varied positively with %OA*’* (rho = 0.978), *p* < 0.001 for all, in line with the explanations given above for the correlation outcomes shown in Figure 1.

#### Computer Experiments to Study the Effect of Altering Ranges upon Associations between Relative Amounts of OA’, EPA’ and AA’

**Figure 3:**
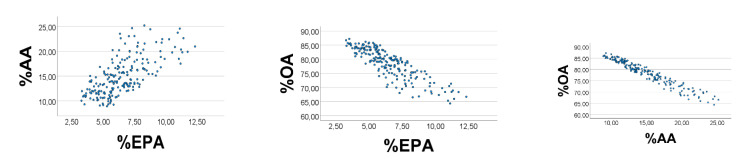
Effect of narrowing the OA*’* range upon associations between relative amounts of EPA*’*, AA*’*, and OA*’*, in a computer experiment. S = OA*’* + EPA*’* + AA*’*. RANDOM numbers (*n* = 200) were used in lieu of true values. Ranges of EPA*’*, AA*’*, and OA*’* were 0.1–0.2, 0.3–0.4, and 1–3, respectively. %EPA*’* vs. %AA*’*: rho = 0.697; %OA*’* vs. %EPA*’* (%AA*’*): rho = −0.852 (−0.964), *p* < 0.001 for all. Note: In the figure we have omitted the random number mark (*‘*) on the variables.

As explained above, increasing (decreasing) the levels of OA, and decreasing (increasing) the levels of AA and EPA, should improve (make poorer) the associations between relative amounts of the variables. For example, if narrowing the OA- range towards the lower limit, we should expect poorer scatterplots and correlation coefficients; this effect should be even stronger if also broadening the ranges of EPA and AA. Conversely, if broadening the OA- range, and narrowing ranges of EPA and AA, we should expect improved scatterplots. With random numbers, we accordingly first narrowed the OA’-range, to be 1–3, while keeping the physiological ranges of EPA*’* and AA*’*. As shown in Figure 3, the associations became somewhat poorer, i.e., %AA*’* vs. %EPA*’*: rho = 0.697; %OA*’* vs. %EPA*’* (%AA*’*): rho = −0.852 (−0.964), *p* < 0.001 for all (*n* = 200).

**Figure 4:**
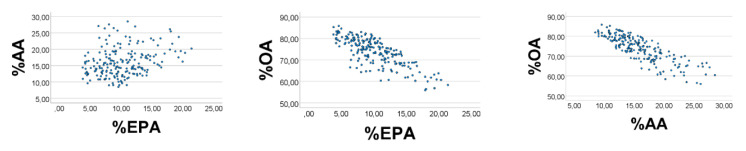
Effect of narrowing the OA*’* range, and broadening ranges of EPA*’* and AA*’*, upon associations between their relative amounts, in a computer experiment. S = OA*’* + EPA*’* + AA*’*. RANDOM numbers (*n* = 200) were used in lieu of true values. Ranges of EPA*’*, AA*’*, and OA*’* were 0.1–0.4, 0.3–0.5, and 1–3, respectively. %EPA*’* vs. %AA*’*: rho = 0.697; %OA*’* vs. %EPA*’* (%AA*’*): rho = −0.852 (−0.964), *p* < 0.001 for all. Note: In the figure we have omitted the random number mark (*‘*) on the variables.

We next broadened the EPA’ range to 0.1–0.4, and the AA’ range to 0.3–0.5, and narrowed the range of OA*’* to 1-3. The correlation outcome became poorer (Figure 4), i.e., %EPA*’* vs. %AA*’*: rho = 0.266; %OA*’* vs. %EPA*’* (%AA*’*): rho = −0.733 (−0.832), *p* < 0.001 for all (*n* = 200).

**Figure 5:**
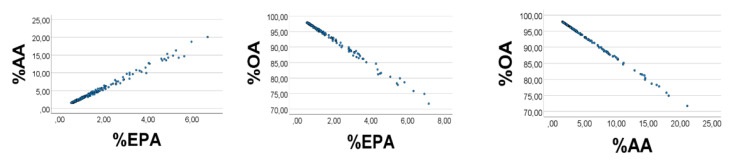
Effect of broadening the OA*’* range, and narrowing ranges of EPA*’* and AA*’*, upon associations between their relative amounts, in a computer experiment. S = OA*’* + EPA*’* + AA*’*. RANDOM numbers (*n* = 200) were used in lieu of true values. Ranges of EPA*’*, AA*’*, and OA*’* were 0.10–0.12, 0.30–0.33, and 1–20, respectively. %EPA*’* vs. %AA*’*: rho = 0.989; %OA*’* vs. %EPA*’* (%AA*’*): rho = −0.994 (−0.999), *p* < 0.001 for all. Equation of the %AA*’* vs. %EPA*’* regression line was %AA*’* = 2.84 (0.02) %EPA*’* + 0.02 (0.04). Note: In the figure we have omitted the random number mark (*‘*) on the variables.

We finally broadened the OA*’* range to 1–20, and narrowed ranges of EPA*’* (AA*’*) to 0.10–0.12 (0.30–0.33). The scatterplots improved appreciably (Figure 5), i.e., %EPA*’* vs. %AA*’*: rho = 0.989; %OA*’* vs. %EPA*’* (%AA*’*): rho = −0.994 (−0.999), *p* < 0.001 for all (*n* = 200). Equation of the %AA*’* vs. %EPA*’* regression line (SE in parentheses) was %AA*’* = 2.84 (0.02) %EPA*’* + 0.02 (0.04). The slope estimate using the AA*’*/EPA*’* ratio was 0.32/0.11 = 2.9, which is not far from the value obtained from the regression line.

These results strongly suggest that distributions (ranges) of the variables govern the correlations between their relative amounts, i.e., the associations are distribution-dependent correlations.

### 3.10. %OA vs. Other Eicosanoid (Docosanoid) Precursor Percentages

Ranges of other eicosanoid (docosanoid) precursor fatty acids in chicken breast muscle were narrow relative to the OA range [28]. For example, the ranges (g/kg wet weight) of DPA (22:5 n3), DHA (22:6 n3) and DGLA (20:3 n6) were 0.2–0.4; 0.1–0.3; and 0.06–0.11, respectively. We should expect, accordingly, that percentages of all these precursor fatty acids were positively associated, as was confirmed with the true values [27]. However, also when using surrogate, random numbers in lieu of the measured values, we were able to largely achieve the same correlation outcomes, if ranges of the random numbers were like the true ones [27]. As discussed below, we suggest that there might possibly be “intended ranges” of fatty acids, caused by evolutionary selection.

### 3.11. Considering Published Data from Human Sera

We utilized reported data [30] from sera of Canadian Caucasians (*n* = 287), and computed S = OA*’* + AA*’* + EPA*’*, based upon random numbers, generated in lieu of the reported mean (SD) values (µmol/L), i.e., 1323 (466) for OA, 402 (129) for AA, and 39 (27) for EPA. We found that %OA*’* correlated negatively with %AA*’* (%EPA*’*): rho = −982 (−0.527), *p* < 0.001, *n* = 200. There was a weak positive association between %AA*’* and %EPA*’*, rho = 0.198 (*p* = 0.005).

**Figure 6:**
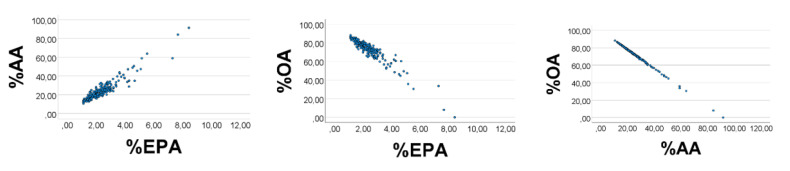
Associations between relative amounts of EPA*’*, AA*’*, and OA*’*, in a computer experiment using RANDOM numbers in lieu of fatty acid values reported in human sera [31]. S = OA*’* + EPA*’* + AA*’*. RANDOM numbers (*n* = 200) with true mean values were used, but with variabilities like the ones found in a homogeneous chicken population (33), i.e., CV being 44% for OA, and 10% for AA and EPA. The mean (SD) values used were: 1323 (582) for OA*’*, 402 (40) for AA*’*, and 39 (4) for EPA*’*. %AA*’* vs. %EPA*’*: rho = 0.887; %OA*’* vs. %AA*’* (%EPA*’*): rho = −0.999 (−0.905), *p* < 0.001 for all, *n* = 200. Note: In the figure we have omitted the random number mark (*‘*) on the variables.

The previous results from chicken muscle indicated that variability was the crucial point to explain associations between relative amounts. To compare better the human sera results with corresponding ones found in the homogeneous chicken population, we repeated the calculations, using the reported mean values, but applying variabilities found in chickens, i.e., coefficient of variation being 44% for OA, and 10% for AA and EPA. The correlation outcome improved (Figure 6); %OA*’* vs. %AA*’* (%EPA*’*): −0.999 (−0.905); %AA*’* vs. %EPA*’*: 0.887, *p* < 0.001 for all, *n* = 200. %EPA*’* and %AA*’* had strong positive skewness, i.e., 3.6, and 3.1, respectively; %OA*’* had strong negative skewness: −3.6 (SE = 0.17 for all).

Quartiles of the %EPA*’*, %AA*’*, and %OA*’* distributions were 1.7, 2.2, 3.0; 17.6, 23.1, 30.3; and 66.6, 74.8, 80.5%, respectively. Thus, the strong inverse relationship between %OA*’* and %AA*’* is in keeping with the equation %OA*’* = −%AA*’* + (100 − %EPA*’*) which would approach %OA*’* = −%AA*’* + 100, with small %EPA*’* values. However, we also found a negative %OA*’* vs. %EPA*’* association, albeit with a poorer scatterplot than that found for %OA*’* vs. %AA*’*. The equation %OA*’* + %AA*’* + %EPA*’* = 100 may be written %OA*’* = −%EPA*’* + (100 − %AA*’*). However, the relatively high %AA*’* values are not in favor of simplifying the equation to %OA*’* = −%EPA*’* + 100, which could have explained the %OA*’* vs. %AA*’* relationship.

**Figure 7:**
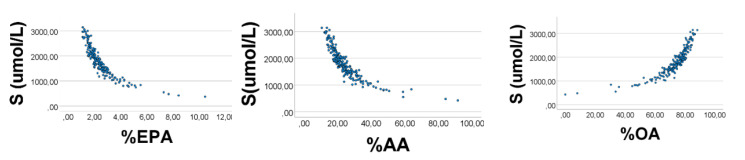
Computer experiment to study associations between S and relative amounts of EPA*’*, AA*’*, and OA*’*, based upon results in sera of humans [31], where S = OA*’* + EPA*’* + AA*’*. RANDOM numbers (*n* = 200) were made in lieu of true values, however with the reported mean values, but using variabilities like those found in chicken muscle [32]. Spearman*’*s rho for S vs. %AA*’* (%EPA*’*): = −0.941 (−0.945); S vs. %OA*’*: rho = 0.951, *p* < 0.001 for all, *n* = 200. Note: In the figure we have omitted the random number mark (*‘*) on the variables.

However, the associations between S and OA (AA, EPA) percentages of S may explain the correlation outcomes. Since we have given AA*’* and EPA*’* narrow ranges relative to that of OA*’*, we should expect S to relate inversely to %EPA*’* and %AA*’*, but positively to %OA*’*, as explained above. As shown in Figure 7, S did correlate negatively with % EPA*’* (%AA*’*), rho = −0.945 (−0.941), and positively with %OA*’* (rho = 0.951), *p* < 0.001 for all; *n* = 200.

We additionally studied associations between relative amounts of substitute, random values for OA, AA, and EPA, using uniform distribution of the random numbers, made from ranges. As expected, the correlation outcomes were as those found with normal distribution of the random numbers, if corresponding variabilities were the same (results not shown).

### 3.12. The Denominator

When computing fractions (percentages) in the present work, we used sum (S) of OA*’*, AA*’*, and EPA*’* in the denominator. It might be questioned whether correlations between percentages would change if S included more of the fatty acids. In general, with a large number of positive scale variables (A, B, C……), their sum is S = A + B + C……, giving the equation: %A + %B + %C + ……= 100, which may be simplified to apparently involve 3 variables: %A + %B + %R = 100, where R is the sum of all variables, except A and B. If both of these latter variables have low ranges and low numbers relative to R, we should expect that %A and %B correlate positively, as explained above. Thus, with this requirement fulfilled, the positive %A vs. %B association should prevail if broadening the R range by including more variables in the denominator. This reasoning was verified in our previous computer experiments.

Computer test: Above, we summed random numbers representing OA, AA, and EPA only; their ranges being 1–9, 0.3–0.4, and 0.1–0.2, respectively. In a repeat of this experiment, we obtained %AA*’* vs. %EPA*’*: rho = 0.867, *p* < 0.001, *n* = 200. Equation of the regression line was %AA’ = 1.78 (0.07) %EPA’ + 0.98 (0.15). We then included altogether 12 fatty acids in the denominator, i.e., the total range of the fatty acids was increased to 3–15 (Reference 33). The %AA*’* vs. %EPA*’* association did not change much: rho = 0.856, *p* < 0.001, *n* = 200. Equation of regression line changed to be %AA’ = 1.96 (0.09) %EPA’ + 0.85 (0.18). This outcome seems in favor of using the *“*three-variable approach*”*.

### 3.13. Major Points and Suggested Interpretations

Our present and previous [25,26,27,28,29] studies suggest that the particular ranges of OA, AA, and EPA could make %OA to be negatively associated with %AA, as a mathematical consequence of the ranges.

Additionally, this negative association should improve when increasing the OA concentration (presumably diet-related). Furthermore, high OA levels seem to improve the positive association between %AA and %EPA, and also improve the relationship between %AA and other eicosanoid (docosanoid) precursor percentages. All of these associations could be physiologically advantageous, serving to obtain a proper balance between the powerful eicosanoids and docosanoids, for example between those derived from AA and EPA [11,18,33,34].

Interestingly, when using the measured mean values of OA (AA, EPA) in human sera [30], but applying variabilities as found in chickens, the associations between relative amounts of the fatty acids in human sera were qualitatively like corresponding ones found in chicken breast muscle. Moreover, the correlation outcomes were as expected, mathematically. Notably, it is not justified to conclude from the present results because we used random numbers in lieu of the measured values. Furthermore, when working with the reported data from human sera, we applied hypothetical ranges in the computer experiments. Nevertheless, the analyses show that, with three scale variables, where two of them have narrow ranges relative to a third variable, we should find that relative amounts of the low-number variables correlate positively. In contrast, percentage of the high-number variable should correlate negatively with percentages of the two low-number variables.

Furthermore, the results demonstrate strong effects of altering the ranges upon associations between relative amounts of positive scale variables, having ranges like the fatty acids under investigation.

Biological variables exist generally within particular ranges, presumably developed because of evolutionary selection. Examples could be body temperature, heart rate, blood pressure, organ sizes, and amounts of many tissue and blood factors, such as glucose, lipoproteins, and fatty acids. In laboratory medicine, the “normal range” of a variable is regularly defined as the mean value ± 2SD, based upon data found in healthy subjects.

Variation is a central concept in statistics, being measured by, e.g., range, interquartile range, and standard deviation. The spread of a biological variable may in general be categorized as true biological, pre-analytic, and analytic. Additionally, variation may be divided into common cause variation and assignable variation [32]. The latter type implies unexpected large variation, caused by for example problems with the supply of water, food, electricity, or computer crash, traffic accidents, and coronavirus infection. Common cause variation, as well as the assignable type, are considered to be negative, and all efforts should be made to reduce or eliminate these types of variations, e.g., to improve accuracy, performance, and productivity in industrial processes, including those in healthcare.

The present work raises the question of whether observed ranges (including place on the scale) of some types of biological variables might be considered differently, i.e., to represent a wanted variation, one that metabolism strives to achieve. In other words, we raise the question of whether there might exist biologically intended, advantageous variation-going from lower to upper limits, and presumably developed through evolutionary selection. During this selection, the necessary regulatory mechanisms should have been developed as well, to obtain the intended limits. Of course, the regulatory processes governing these limits would be subject to common cause and assignable variation. We hypothesize that ranges of tissue and blood fatty acids could be examples of intended ranges in biology.

It is well known that there is metabolic turnover in tissues and blood. As time goes by, amounts of many components may increase and decrease, because of the balance between synthesis and degradation, making the biological amounts to exist within metabolically controlled upper and lower limits. We may picture the dynamic variations of, e.g., fatty acid amounts, in blood and tissues, to be like ponds with inlets and outlets. By strictly controlling the inlet/outlet balance, the pond might vary within certain intended limits only, to obtain *“*fatty acid ponds*”* of very varying sizes.

One example of metabolic turnover concerns muscle triglycerides (TG). Fatty acids enter blood from the intestines, become bound to serum albumin, and are then taken up by muscle, where the fatty acids are partly incorporated into TG, and partly utilized for energy production [35]. At the same time, TGs are broken down by lipases, released to the blood, where they are bound to serum albumin, and may, again, be taken up and utilized in many tissues (e.g., muscle, fat, liver). Thus, the balance between TG synthesis and degradation will determine whether the muscle triglyceride concentration increases or decreases, or appear unchanged. In a trial in humans [35], 50–60% of blood fatty acids were esterified to TG, and 30–40% oxidized, during a period. The fractional TG synthesis rate was estimated to be 3.4%·h^−1^. Since the TG concentration did not change during this experiment, the authors concluded that synthesis balanced breakdown.

As shown mathematically in the present article, percentages of variables, such as fatty acids, should correlate positively or negatively, on the conditions that their concentrations exist within particular ranges. We suggest that these ranges might be considered to be intended ones. Conceivably, the measured ranges will also reflect variations related to time of sampling, food intake, physical activity, and so forth. Additionally, errors could be attributed to other external factors, related to sampling, measurement, information bias, and environment in general.

The many causes of error could make it difficult to find the suggested biological, intended variability, e.g., of particular fatty acid concentrations, and we should regularly study frequency distributions and scatterplots, to find outliers. Thus, very low external noise (common cause variation) and no assignable (unexpected) variability would be required to detect the suggested true, intended ranges. However, as shown mathematically in this article, distribution-dependent correlations should exist, if the variables in question do have the required ranges. In this regard, we emphasize that the chicken population referred to in this work was very homogeneous, genetically and environmentally, thereby offering an excellent opportunity to evaluate how the suggested intended ranges might influence associations between relative amounts of fatty acids [25,26,27,28,29]. Since the mathematical rules giving distribution-dependent correlations are general ones, they should apply to any unit system in nature. Biochemical mechanisms behind the intended concentration ranges could, in general, involve regulation of enzyme synthesis, allosteric regulation of enzyme activities, feedback regulation, and interconversion between phosphorylated and dephosphorylated forms of key enzymes. In this context, enzymes regulating formation and catabolism of OA, AA, and EPA are crucial for obtaining the particular ranges, important enzymes being desaturases and elongases (ELOVL family). Diet, physical activity, and lifestyle factors in general might influence activities and/or amounts of the enzymes and, subsequently, the fatty acid amounts/concentrations.

Thus, evolution might have “understood” that if selecting particular ranges (“intended ranges”) of some fatty acids, then the percentages of AA and EPA must be positively associated, mathematically. Presumably, the purpose of this evolutionary selection is to ensure a proper balance between metabolites having antagonistic actions (e.g., eicosanoids derived from AA vs. those from EPA). Practically, this mathematical phenomenon could imply that increased intake of foods rich in oleic acid, such as olive oil, would improve the balance between AA and EPA (and their eicosanoids).

Possibly, a novel beneficial effect of oleic acid could exist, in addition to the many other positive effects reported previously [1,2,3,4,5,6,7,8,9,10,31,36].

### 3.14. Limitations of the Study

In this work, we conducted computer experiments based upon reported values of OA, AA, and EPA, measured in chicken muscle and in human sera. Future studies should investigate whether ranges might govern associations between percentages of other fatty acids as well, and comparable studies should be done in other species, including humans. Additionally, more work should be done to clarify the suggested concepts of intended ranges and distribution-dependent correlations.

## 4. Conclusions

We suggest that ranges of OA, AA, EPA, and other eicosanoid (docosanoid) precursor fatty acids could be “*intended ranges”*, appearing through evolutionary selection. Mathematically, the intended ranges seem to govern associations between relative amounts of fatty acids, e.g., giving *distribution-dependent correlations,* DDCs (positive and negative) between OA, AA, and EPA percentages. Practically, the mathematical phenomenon of DDC could imply that increased intake of foods rich in oleic acid, such as olive oil, might improve the balance between AA and EPA (and their eicosanoids). 

## Data Availability

In the current computer experiments, we used random numbers only.
